# Supplementary material for: Error-driven upregulation of memory representations
Source: Commun Psychol. 2025 Jan 30;3:17. doi: 10.1038/s44271-025-00199-5 (PMC11782628; doi:10.1038/s44271-025-00199-5)
Supplement: Supplementary file 2 — Supplementary Information [file 44271_2025_199_MOESM2_ESM.pdf]

Supplementary Information for the Manuscript:

Error-driven upregulation of memory representations

Alexander Weuthen[1,2,3,4,*], Hans Kirschner[1], Markus Ullsperger[1,3,4,5]

1 Institute of Psychology, Otto-von-Guericke-University Magdeburg, Magdeburg, Germany

2 Department of Psychiatry and Psychotherapy, Jena University Hospital/Friedrich-Schiller-University, Jena, Germany

3 German Center for Mental Health (DZPG), partner site Halle-Jena-Magdeburg, Germany

4 Center for Intervention and Research on adaptive and maladaptive brain Circuits underlying mental health (C-I-R-C), Halle-Jena-Magdeburg, Germany

5 Center for Behavioral Brain Sciences, Magdeburg, Germany

* Corresponding Author: Alexander Weuthen, Klinik für Psychiatrie und Psychotherapie, Universitätsklinikum Jena, Philosophenweg 3, 07743 Jena, Germany; Phone: +49 (0)3641 9390230; E-Mail: Alexander.Weuthen@uni-jena.de

Supplementary Figures

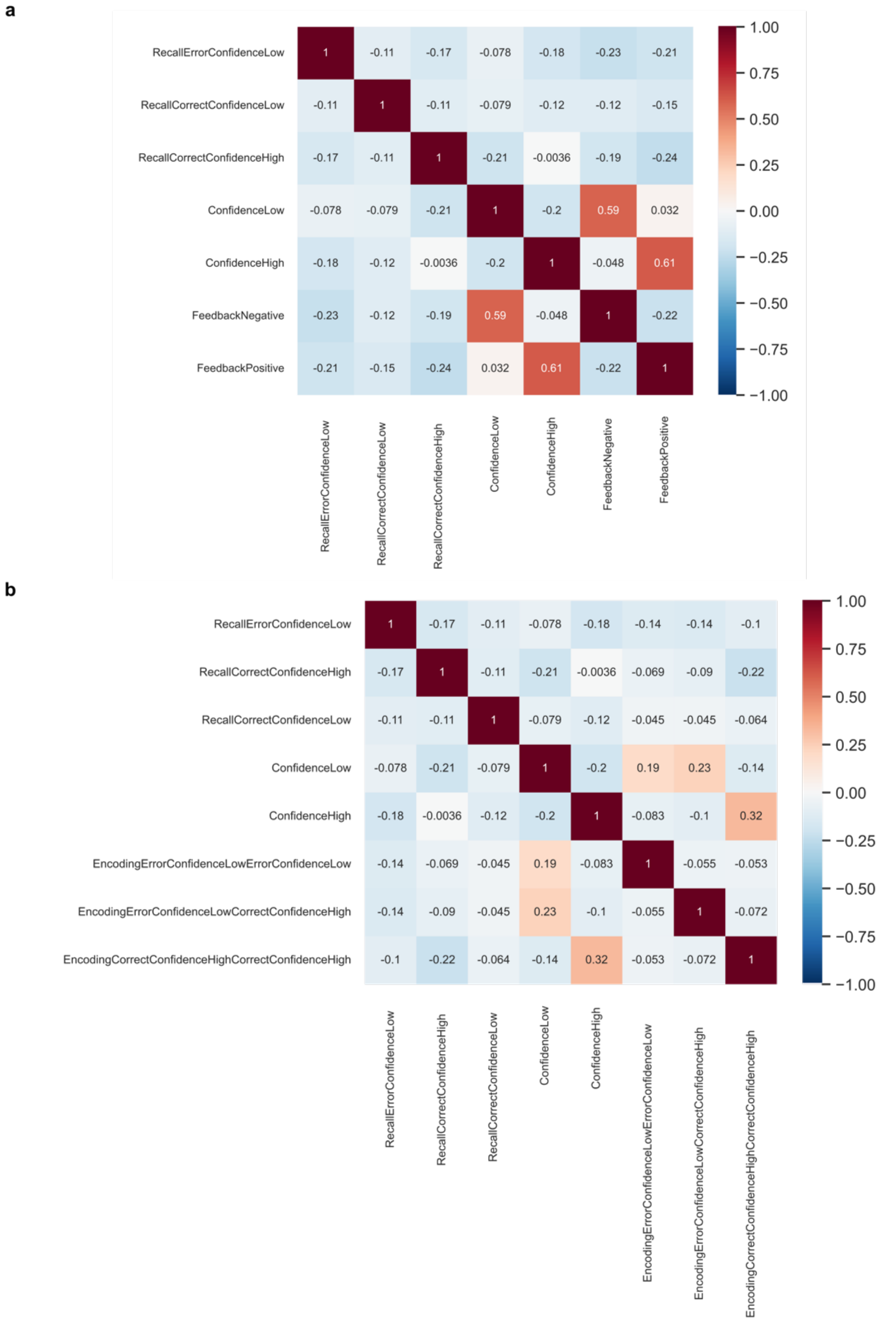

Supplementary Figure 1 | Correlation matrix of the design matrix regressors. The matrix shows the average Pearson correlation between different trial types in the general linear model for **a,** error-monitoring processes and **b,** post-error subsequent memory effect for all 30 participants (15 male, 15 female).

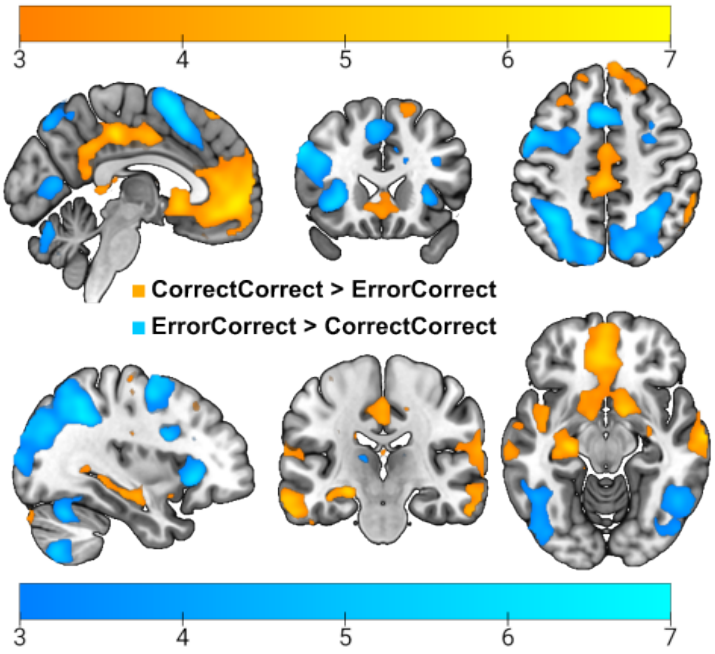

Supplementary Figure 2 | General linear model results differentiating between Correct$_{\text{ConfidenceHigh}}$Correct$_{\text{ConfidenceHigh}}$ and Error$_{\text{ConfidenceLow}}$Correct$_{\text{ConfidenceHigh}}$ trials. Increased hemodynamic responses (orange) are found in the hippocampus and medial frontoparietal default mode network regions, suggesting neurophysiological underpinnings of reward-based reconsolidation processes. Decreased hemodynamic responses (blue) are found in nodes of the midcinguloinsular salience/ ventral attention network such as anterior insula and posterior medial frontal cortex, suggesting regions involved in initial error-driven association learning success.

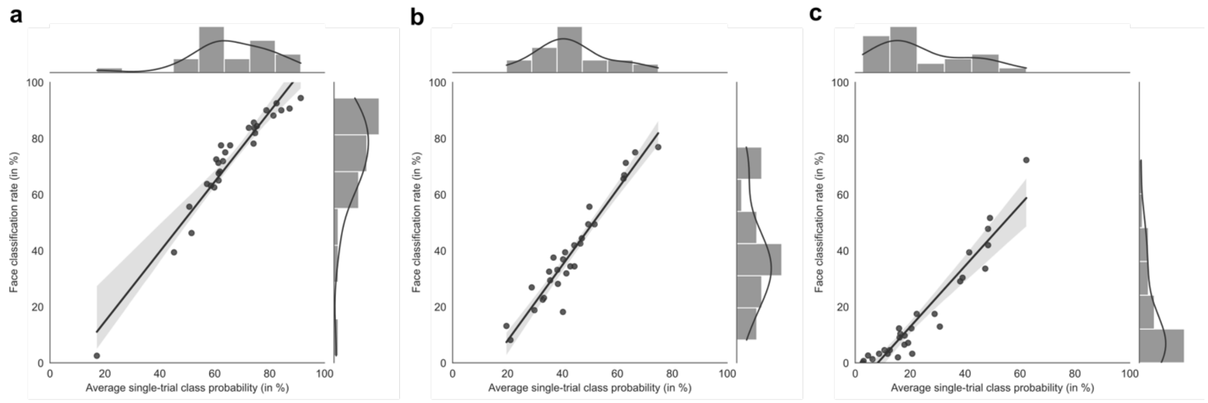

Supplementary Figure 3 | Relationship between the average rate of face predictions per participants and average single-trial face-class probability. To validate the cross-classification-based single-trial stimulus evidence parameter, inter-individual associations were assessed (n = 28, two participants were excluded from cross-classification analyses based on low localizer task performance). Cross-classification prediction rates and average class probability showed a strong association (Spearman correlation > .90) during all three epochs, **a,** recall, **b,** encoding and **c,** inter-trial-interval. Confidence intervals represent a 95% range based on 1000 times bootstrapping.

Supplementary Tables

Supplementary Table 1 | Post-hoc analysis of block differences in the feedback-based association learning task using Tukey's honestly significant differences.

Comparison	Statistic	p-value	Lower CI_{95}	Upper CI_{95}
Block 1 and 2	-17.800	< .001	-22.436	-13.164
Block 1 and 3	-24.600	<.001	-29.236	-19.964
Block 1 and 4	-26.833	< .001	-31.469	-22.197
Block 2 and 1	17.800	<.001	13.164	22.436
Block 2 and 3	-6.800	.001	-11.436	-2.164
Block 2 and 4	-9.033	<.001	-13.669	-4.397
Block 3 and 1	24.600	<.001	19.964	29.236
Block 3 and 2	6.800	.001	2.164	11.436
Block 3 and 4	-2.233	.593	-6.869	2.403
Block 4 and 1	26.833	< .001	22.197	31.469
Block 4 and 2	9.033	< .001	4.397	13.669
Block 4 and 3	2.233	.593	-2.403	6.869

Supplementary Table 2 | Implicit memory error evidence. Significant clusters during failed recall ($Error_{LowConfidence}$ > $Correct_{HighConfidence}$) in the feedback-based association learning task for voxels with a p_{FDR} < .05 and clusters of at least 5 continuous voxels according to automated anatomical labeling (AAL) atlas. Clusters without specified cluster size represent subclusters of above specified regions.

Region	X	Y	Z	Peak statistic	Cluster size
Occipital Inf R	27	-90	-9	6.298	65112
Lingual L	-36	-85	-16	6.149	
Occipital Inf L	-23	-96	-5	5.817	
Lingual L	-10	-92	-16	5.780	
Frontal Inf Tri R	51	28	20	5.167	6995
Frontal Inf Oper R	55	17	36	3.762	
unspecified in AAL	-1	17	1	4.961	11957
Caudate R	11	-2	14	4.797	
unspecified in AAL	-18	-24	20	4.694	
unspecified in AAL	0	-37	5	4.583	
Fusiform L	-29	-48	-9	4.909	2257
Frontal Inf Tri L	-45	28	23	4.867	6165
Frontal Inf Tri L	-51	22	29	4.675	
Precentral L	-38	4	34	3.762	
Cingulum Mid R	5	22	40	4.299	3002
Supp Motor Area L	-7	19	45	3.910	
Frontal Sup Medial R	5	33	42	3.297	
Supp Motor Area R	9	15	56	3.090	
unspecified in AAL	-23	-46	20	4.103	841
unspecified in AAL	-21	-57	25	3.627	
Cerebelum 10 L	-23	-35	-42	3.928	883
Cerebelum 4 5 L	-25	-28	-31	3.451	
Temporal Pole Sup R	66	6	-1	3.818	191
Supp Motor Area L	-1	22	67	3.595	202
Precentral R	18	-24	78	3.535	330
Temporal Sup R	49	-8	-3	3.530	489
Temporal Sup R	47	-6	-12	3.000	
unspecified in AAL	38	0	-14	3.496	308
Hippocampus R	36	-19	-9	3.446	223
Frontal Mid R	27	13	56	3.392	447
Heschl R	51	-17	9	3.343	404
Frontal Sup L	-23	66	7	3.334	255
unspecified in AAL	22	-32	53	3.322	95
unspecified in AAL	14	-15	23	3.290	117
Caudate R	20	-21	20	3.286	234
unspecified in AAL	-1	50	51	3.266	63
unspecified in AAL	29	-26	-1	3.266	117

Paracentral Lobule R	3	-32	73	3.089	53
Supp Motor Area R	9	8	69	3.083	63
Calcarine L	-14	-54	12	3.069	53
Temporal Sup R	69	-13	7	3.009	63
unspecified in AAL	27	-26	29	2.949	53
Heschl L	-45	-15	5	2.887	63

Supplementary Table 3 | Internal memory error evidence. Increased hemodynamic responses for the selection of low compared to high recall certainty during confidence selection in the feedback-based association learning task, for voxels with a $p_{FDR} < .05$ and clusters of at least 5 continuous voxels according to automated anatomical labeling (AAL) atlas. Clusters without specified cluster size represent subclusters of above specified regions.

**Region**	**X**	**Y**	**Z**	**Peak statistic**	**Cluster size**
Frontal Mid R	36	48	20	5.774	33338
Frontal Sup R	22	13	65	5.572	
Frontal Mid R	42	35	29	4.952	
Frontal Mid R	44	28	42	4.853	
Precuneus R	9	-70	45	5.291	18506
Precuneus L	-7	-68	49	5.056	
Precuneus R	5	-70	56	4.983	
Precuneus R	14	-61	29	4.779	
SupraMarginal R	55	-46	29	5.038	20955
Parietal Inf R	51	-41	53	4.685	
Parietal Sup R	38	-61	56	4.397	
Parietal Inf R	47	-57	49	4.301	
Frontal Inf Tri L	-45	28	27	4.933	13416
Frontal Inf Tri L	-34	24	29	4.137	
Frontal Mid L	-38	55	12	3.951	
unspecified in AAL	-38	59	1	3.762	
Cingulum Mid R	3	35	36	4.866	3652
Supp Motor Area L	-3	19	47	4.369	
Frontal Sup Medial R	3	41	51	2.857	
Temporal Mid R	69	-21	-3	4.571	5292
Temporal Mid R	66	-30	-5	4.219	
Temporal Inf R	55	-30	-23	3.694	
Temporal Mid R	64	-50	-5	3.571	
Parietal Inf L	-51	-43	49	4.541	9040
Parietal Inf L	-32	-74	49	4.480	
Parietal Inf L	-36	-54	38	3.956	
Angular L	-43	-61	47	3.636	
unspecified in AAL	-25	-87	-45	3.835	489
unspecified in AAL	-36	-83	-45	2.906	
unspecified in AAL	-5	-32	-16	3.776	170
unspecified in AAL	0	-24	27	3.610	500
Cingulum Mid R	9	-26	31	2.938	
Precuneus R	11	-37	3	3.579	383
unspecified in AAL	0	-30	7	2.922	
Insula L	-29	26	-5	3.481	468

Supp Motor Area L	-12	6	67	3.439	1331
Frontal Mid L	-21	11	62	3.218	
unspecified in AAL	0	-13	-27	3.328	149
Frontal Sup R	22	59	29	3.315	117
unspecified in AAL	9	-26	-18	3.168	106
unspecified in AAL	-25	37	-25	3.151	138
Frontal Sup R	20	70	3	3.052	63
unspecified in AAL	-23	-26	29	3.036	85
Frontal Mid Orb L	-18	50	-16	2.978	138
unspecified in AAL	-18	-52	27	2.916	74

Supplementary Table 4 | External memory error evidence. Hemodynamic responses increased for negative compared to positive feedback in the feedback-based association learning task, for voxels with a $p_{FDR} < .05$ and clusters of at least 5 continuous voxels according to automated anatomical labeling (AAL) atlas. Clusters without specified cluster size represent subclusters of above specified regions.

Region	X	Y	Z	Peak statistic	Cluster size
Occipital Mid R	36	-76	34	7.618	187085
Parietal Inf L	-34	-54	45	7.360	
Occipital Mid L	-27	-70	25	6.818	
Parietal Inf L	-27	-79	42	6.782	
Insula R	31	26	-1	6.958	33605
Frontal Sup R	29	4	60	6.312	
Frontal Inf Oper R	51	11	29	5.522	
Precentral R	27	-4	47	5.077	
Insula L	-29	24	1	6.881	60416
Frontal Inf Tri L	-49	26	27	6.806	
Supp Motor Area R	5	17	51	6.804	
Frontal Mid L	-25	0	58	6.694	
Cerebelum 6 R	11	-74	-25	6.314	13459
Cerebelum 7b L	-29	-70	-47	6.020	
Cerebelum Crus1 L	-7	-74	-25	5.194	
Cerebelum Crus2 R	5	-81	-36	4.717	
Cerebelum 9 R	9	-52	-51	5.677	2214
Cerebelum 8 R	29	-72	-49	5.070	3322
unspecified in AAL	-1	-32	-25	3.799	819
ParaHippocampal L	-29	-43	-7	3.778	393
unspecified in AAL	-3	6	-42	3.546	85
Cerebelum 8 R	31	-41	-47	3.468	170
Lingual R	5	-85	-7	3.170	181
Frontal Sup Orb R	25	52	-7	3.151	351
unspecified in AAL	25	37	-25	3.080	117
unspecified in AAL	5	-6	-3	3.016	106
unspecified in AAL	27	33	-27	2.913	53
unspecified in AAL	-25	33	-27	2.890	223
Fusiform R	36	-15	-36	2.870	340
Fusiform R	33	-6	-34	2.727	
unspecified in AAL	-16	8	9	2.775	223
Fusiform L	-40	-32	-20	2.619	95
Temporal Sup R	44	-28	3	2.564	85
Frontal Mid Orb L	-38	44	-3	2.549	95

Supplementary Table 5 | The post-error subsequent memory effect. The table shows regions with increased hemodynamic responses for later recall success during the encoding epochs which followed memory errors ($Error_{LowConfidence}Correct_{HighConfidence}$ > $Error_{LowConfidence}Error_{LowConfidence}$), for voxels with a p_{FDR} < .05 and clusters of at least 5 continuous voxels according to automated anatomical labeling (AAL) atlas. Clusters without specified cluster size represent subclusters of above specified regions.

Region	X	Y	Z	Peak statistic	Cluster size
Supp Motor Area L	-3	0	71	5.370	2299
Supp Motor Area L	-10	4	73	5.149	
Supp Motor Area L	-1	13	69	4.387	
Occipital Mid L	-27	-79	40	4.374	191
Frontal Inf Tri L	-49	28	12	4.323	372
Frontal Inf Tri L	-56	24	23	3.799	
Fusiform L	-40	-54	-18	4.294	1224
Temporal Inf L	-49	-50	-18	4.192	
unspecified in AAL	-62	-63	-1	4.293	692
Temporal Mid L	-43	-74	20	4.250	202
Frontal Inf Oper L	-56	8	9	4.040	628
Frontal Inf Tri L	-54	15	5	3.845	
Occipital Inf R	47	-72	-14	3.991	340
Temporal Inf R	51	-65	-12	3.912	
unspecified in AAL	-51	26	-20	3.979	106
unspecified in AAL	-47	-35	-1	3.945	63
Cerebelum Crus1 R	49	-61	-25	3.943	170
Temporal Pole Sup L	-27	13	-27	3.855	53
Precentral L	-54	0	49	3.830	298
Fusiform L	-25	-57	-16	3.748	74

Supplementary Table 6 | Increased hemodynamic responses for repeated correct (Correct$_{\text{ConfidenceHigh}}$Correct$_{\text{ConfidenceHigh}}$) compared to initial correct learning success (Error$_{\text{ConfidenceLow}}$Correct$_{\text{ConfidenceHigh}}$) during encoding epochs in the feedback-based association learning task. Effects are shown for voxels with a p_{FDR} < .05 and clusters of at least 5 continuous voxels according to automated anatomical labeling (AAL) atlas. Clusters without specified cluster size represent subclusters of above specified regions.

Region	X	Y	Z	Peak statistic	Cluster size
Frontal_Med_Orb_L	-1	52	-5	6.542	213471
Cingulum_Mid_L	-3	-28	42	6.186	
Temporal_Mid_R	69	-13	-9	6.048	
Hippocampus_L	-23	-21	-12	5.864	
unspecified in AAL	-58	-59	40	4.217	5164
unspecified in AAL	-27	-92	-27	4.075	4110
unspecified in AAL	-14	-94	-27	4.051	
unspecified in AAL	-25	-90	-40	3.092	
unspecified in AAL	-36	-83	-45	3.072	
Cerebelum_Crus1_R	27	-85	-27	3.968	2321
Cerebelum_Crus2_R	27	-90	-38	3.371	
unspecified in AAL	16	-13	25	3.847	287
Precentral_L	-32	-17	56	3.777	1341
Precentral_L	-36	-17	67	3.627	
Precentral_L	-34	-28	65	2.716	
Calcarine_R	18	-83	3	3.766	330
Frontal_Inf_Orb_L	-38	50	-16	3.732	543
Frontal_Mid_R	49	46	3	3.698	1756
Frontal_Mid_Orb_R	44	52	-7	3.612	
Cerebelum_Crus2_R	47	-57	-45	3.445	170
unspecified in AAL	-67	-46	1	3.427	543
unspecified in AAL	0	-39	-64	3.414	734
unspecified in AAL	-27	30	16	3.372	223
ParaHippocampal_L	-14	-32	-9	3.341	234
Frontal_Mid_R	40	26	42	3.140	489
Hippocampus_R	33	-10	-18	3.081	298
unspecified in AAL	14	-21	-47	3.061	53
Cuneus_R	14	-92	31	3.049	692
Cuneus_L	0	-90	29	2.821	
Cerebelum_Crus1_R	49	-37	-29	3.008	127
unspecified in AAL	9	-13	-38	2.882	159
Temporal_Pole_Sup_L	-36	24	-23	2.803	149
Temporal_Pole_Mid_R	38	22	-40	2.760	117
Temporal_Pole_Mid_R	38	13	-36	2.602	

Precentral_L	-25	-15	76	2.752	53
Postcentral_L	-21	-28	78	2.751	106
Parietal_Sup_L	-23	-43	71	2.571	74

Supplementary Table 7 | Face-selective regions. The table displays regions found to show increased hemodynamic responses for faces compared to houses in the 1-back localizer task, for voxels with a $p_{FDR} < .05$ and clusters of at least 5 continuous voxels according to automated anatomical labeling (AAL) atlas. Clusters without specified cluster size represent subclusters of above specified regions.

Region	X	Y	Z	Peak statistic	Cluster size
Precuneus R	3	-65	38	6.401	22424
Precuneus R	3	-52	20	5.282	
Calcarine L	-14	-79	12	4.483	
Cingulum Post L	-7	-46	31	4.447	
Cerebelum 6 R	44	-46	-27	5.477	3726
Temporal Mid R	53	-65	5	5.246	18357
Temporal Mid R	53	-57	7	5.129	
Temporal Mid R	51	-39	5	4.482	
unspecified in AAL	44	-41	16	4.370	
Frontal Mid R	22	28	40	5.137	4099
Hippocampus R	18	-6	-16	5.115	1054
Frontal Med Orb L	-3	46	-14	5.036	25597
Rectus R	3	44	-18	4.945	
Frontal Med Orb R	0	52	-12	4.921	
Frontal Sup L	-27	66	9	4.582	
Temporal Pole Sup R	40	19	-31	4.873	5717
Temporal Pole Sup R	31	8	-27	4.565	
unspecified in AAL	31	8	-18	4.321	
Temporal Pole Mid R	51	13	-34	3.617	
Temporal Pole Mid L	-45	17	-29	4.720	5206
Temporal Pole Sup L	-32	8	-25	4.486	
Amygdala L	-18	-4	-14	3.924	
unspecified in AAL	-21	0	-7	3.367	
Fusiform L	-40	-52	-23	4.683	1533
Temporal Mid L	-62	-10	-12	4.307	2374
Temporal Sup L	-45	-15	-12	4.125	
Temporal Mid L	-54	-15	-9	4.101	
Temporal Mid L	-54	-4	-18	2.941	
Frontal Inf Tri R	38	28	12	4.048	340
Temporal Mid R	51	-6	-18	4.036	3162
Temporal Sup R	66	-8	-9	3.94	
Temporal Sup R	55	-8	-7	3.638	
Temporal Mid R	64	-2	-18	3.282	
Cingulum Mid L	-1	-19	40	3.966	1693
Cingulum Mid L	0	-4	38	3.156	
unspecified in AAL	0	6	-12	3.956	1341
unspecified in AAL	-51	-74	12	3.861	4525

Temporal Mid L	-49	-59	9	3.619	
Angular L	-49	-68	25	3.312	
unspecified in AAL	-51	-70	38	3.160	
Cerebelum Crus2 L	-7	-85	-40	3.816	585
Parietal Sup R	20	-52	62	3.689	266
unspecified in AAL	16	-43	58	3.111	
Precentral R	27	-17	78	3.642	234
unspecified in AAL	33	-21	73	3.091	
SupraMarginal R	58	-28	20	3.635	947
Cerebelum Crus1 R	49	-74	-38	3.575	298
Cerebelum Crus1 R	40	-79	-36	2.911	
unspecified in AAL	-25	-50	16	3.568	415
unspecified in AAL	-23	-41	18	3.013	
Insula L	-45	8	-5	3.561	681
Frontal Inf Orb L	-45	17	-12	3.372	
Postcentral L	-43	-24	29	3.525	181
Frontal Mid L	-27	26	58	3.502	2587
Frontal Mid L	-25	35	49	3.349	
Frontal Mid L	-21	24	51	3.306	
Frontal Sup L	-14	24	65	3.204	
unspecified in AAL	-21	41	7	3.475	436
Precentral L	-23	-24	80	3.454	170
Postcentral L	-25	-41	60	3.429	212
Thalamus R	11	-30	9	3.400	191
Frontal Sup L	-18	13	67	3.199	266
Supp Motor Area R	7	-15	58	3.197	53
Hippocampus L	-14	-39	5	3.189	63
Parietal Sup L	-23	-48	76	3.146	212
unspecified in AAL	-62	-17	-29	3.143	74
Cerebelum Crus1 R	51	-61	-38	3.114	127
unspecified in AAL	-7	-6	12	3.110	74
Frontal Mid R	25	17	56	3.074	95
Temporal Mid R	69	-37	1	3.033	63
Precentral R	42	-13	67	3.024	85
Frontal Inf Orb L	-36	28	-18	2.996	74
unspecified in AAL	-34	-61	9	2.993	63
Frontal Inf Orb L	-23	28	-16	2.987	95
Cingulum Ant R	3	41	5	2.965	223
Cerebelum 7b R	42	-59	-49	2.857	63

Supplementary Table 8 | House-selective regions. The table shows which regions displayed increased hemodynamic responses for houses compared to faces in the 1-back localizer task, for voxels with a $p_{FDR} < .05$ and clusters of at least 5 continuous voxels according to automated anatomical labeling (AAL) atlas. Clusters without specified cluster size represent subclusters of above specified regions.

**Region**	**X**	**Y**	**Z**	**Peak statistic**	**Cluster size**
Lingual L	-29	-52	-5	7.019	111399
Fusiform R	29	-52	-3	6.809	
Occipital Mid R	36	-85	16	6.527	
Occipital Mid L	-32	-90	12	6.445	
Cerebelum 7b L	-29	-74	-51	4.298	1086
unspecified in AAL	7	-68	-58	4.064	564
Precentral L	-51	4	40	3.937	1788
Frontal Inf Orb L	-29	28	-7	3.767	244
Cerebelum 8 R	27	-72	-51	3.759	457
Temporal Inf R	55	-50	-9	3.577	468
unspecified in AAL	3	-8	-25	3.433	127
unspecified in AAL	-5	-37	-25	3.371	138
unspecified in AAL	-16	30	-31	3.160	74
Cerebelum Crus2 R	5	-76	-36	3.080	63
Temporal Inf R	49	-61	-9	3.025	95
Cerebelum Crus1 L	-56	-57	-34	3.015	74
Supp Motor Area R	9	15	47	2.997	53
Parietal Inf R	44	-37	53	2.879	53

Supplementary Table 9 | Recall-related face processing regions. Significant clusters related to higher evidence for face-processing during single-trial recall epochs in the feedback-based association learning task, for voxels with a $p_{FDR} < .05$ and clusters of at least 5 continuous voxels according to automated anatomical labeling (AAL) atlas. Clusters without specified cluster size represent subclusters of above specified regions.

Region	X	Y	Z	Peak statistic	Cluster size
Cuneus R	14	-96	7	5.563	89794
Occipital Inf L	-23	-92	-5	5.008	
Occipital Inf R	31	-85	-16	4.986	
unspecified in AAL	38	-92	-16	4.944	
Cerebelum 4 5 R	7	-46	-16	4.813	2683
Cerebelum 4 5 L	-5	-52	-14	3.761	
Cerebelum 4 5 L	-7	-48	-3	3.498	
Supp Motor Area R	3	17	49	4.805	3481
Supp Motor Area L	-7	17	49	3.602	
unspecified in AAL	25	37	-25	4.403	745
unspecified in AAL	20	30	-29	3.949	
Frontal Inf Oper L	-38	2	27	4.303	3854
Frontal Inf Tri L	-43	17	27	3.529	
Frontal Inf Oper L	-54	22	31	3.411	
Angular R	27	-59	42	4.262	3907
Parietal Sup R	20	-68	47	3.519	
Parietal Sup R	31	-72	53	3.478	
Parietal Sup R	29	-63	51	3.167	
Cerebelum Crus1 L	-12	-68	-29	4.012	1299
unspecified in AAL	-14	-54	-36	3.174	
Insula R	29	24	-1	3.947	500
unspecified in AAL	18	8	29	3.934	287
Cerebelum 10 R	22	-37	-47	3.869	1181
unspecified in AAL	11	4	1	3.854	255
Parietal Inf L	-38	-54	42	3.852	2108
Thalamus L	-14	-13	5	3.818	181
Cerebelum 8 R	33	-65	-56	3.743	1181
unspecified in AAL	-5	-26	29	3.743	170
Paracentral Lobule R	9	-35	65	3.687	117
Pallidum L	-18	-2	-3	3.623	181
unspecified in AAL	-10	-2	25	3.598	436
Cingulum Ant L	-5	4	27	3.073	
Cerebelum 10 L	-25	-35	-40	3.576	851
Insula L	-32	24	-1	3.530	404
unspecified in AAL	36	17	20	3.524	255

Cerebelum 8 L	-27	-68	-53	3.517	383
unspecified in AAL	-23	-32	14	3.404	85
unspecified in AAL	-27	-32	1	3.347	255
unspecified in AAL	5	-2	23	3.341	53
Caudate L	-18	-19	23	3.273	74
unspecified in AAL	16	-30	-3	3.225	543
unspecified in AAL	5	-30	-1	3.103	
ParaHippocampal R	36	-13	-29	3.207	223
unspecified in AAL	25	-52	-34	3.175	63
Postcentral L	-21	-50	53	3.151	53
unspecified in AAL	-16	28	-9	3.128	53
Frontal Inf Tri R	53	33	25	3.077	340
Caudate L	-7	8	1	3.014	85
Precuneus R	16	-61	27	3.000	85
unspecified in AAL	-1	-30	-18	2.984	53
unspecified in AAL	25	-32	31	2.975	85
Parietal Sup L	-21	-63	53	2.975	149

Supplementary Table 10 | Encoding-related face processing regions. Significant clusters related to higher evidence for face-processing during single-trial encoding epochs in the feedback-based association learning task, for voxels with a $p_{FDR} < .05$ and clusters of at least 5 continuous voxels according to automated anatomical labeling (AAL) atlas. Clusters without specified cluster size represent subclusters of above specified regions.

Region	X	Y	Z	Peak statistic	Cluster size
Precentral L	-38	-4	53	6.245	11915
Frontal Inf Tri L	-43	13	25	4.556	
Frontal Inf Tri L	-49	33	16	2.928	
Vermis 9	-1	-57	-38	6.080	121834
Occipital Mid L	-10	-103	1	5.575	
Occipital Inf R	38	-70	-9	5.345	
Cerebelum 6 L	-40	-52	-25	5.290	
Supp Motor Area R	5	13	47	5.240	4940
Supp Motor Area L	-5	6	60	3.700	
Parietal Sup R	29	-72	51	4.614	17643
unspecified in AAL	31	-50	36	4.523	
Occipital Mid R	36	-68	25	4.214	
Angular R	25	-63	47	4.153	
Parietal Inf L	-34	-50	51	4.301	16504
Parietal Inf L	-38	-54	42	4.238	
Parietal Sup L	-27	-61	45	4.154	
Parietal Sup L	-36	-63	53	4.016	
unspecified in AAL	25	35	-23	4.080	1341
Fusiform R	40	-8	-34	3.949	1235
Cerebelum 9 R	20	-39	-47	3.802	287
unspecified in AAL	-27	35	-25	3.767	383
Temporal Sup R	44	-35	3	3.656	425
Insula L	-34	17	-3	3.517	873
Frontal Inf Tri L	-32	30	1	3.264	
Cerebelum 8 L	-25	-70	-49	3.506	734
unspecified in AAL	25	-26	-1	3.479	617
Frontal Inf Tri R	49	30	16	3.465	2683
Frontal Inf Tri R	42	15	23	3.159	
unspecified in AAL	36	11	20	3.139	
Paracentral Lobule R	9	-39	65	3.386	287
Cerebelum 9 L	-18	-39	-47	3.368	138
unspecified in AAL	16	-4	-9	3.196	106
Temporal Pole Sup L	-25	6	-25	3.195	276
Lingual R	22	-50	-1	3.168	117
Thalamus R	16	-15	14	3.146	223

unspecified in AAL	-5	-46	-23	3.126	138
Hippocampus L	-14	-6	-14	3.114	85
Precuneus L	-7	-79	47	3.073	159
unspecified in AAL	-40	-21	-42	3.006	53
Supp Motor Area R	5	-19	53	2.986	63
Calcarine R	16	-72	9	2.958	170
unspecified in AAL	29	26	-3	2.901	212
Frontal Mid R	38	-2	58	2.796	95
Paracentral Lobule L	-1	-35	62	2.730	85
unspecified in AAL	5	6	-31	2.724	53
unspecified in AAL	-5	-30	27	2.709	53
Precentral R	33	-2	45	2.658	63

Supplementary Table 11 | Inter-trial-interval-related face processing regions. Significant clusters related to higher evidence for face-processing during single-trial fixation epochs, which may have been used by the participants for the inter-trial-interval in the feedback-based association learning task, for voxels with a $p_{FDR} < .05$ and clusters of at least 5 continuous voxels according to automated anatomical labeling (AAL) atlas. Clusters without specified cluster size represent subclusters of above specified regions.

Region	X	Y	Z	Peak statistic	Cluster size
Cerebelum 6 L	-36	-57	-23	5.999	129266
Occipital Inf R	36	-70	-9	5.752	
unspecified in AAL	-7	-107	5	5.483	
Occipital Mid L	-16	-96	3	5.119	
Postcentral R	11	-37	69	4.749	1022
Postcentral R	18	-32	73	3.146	
Paracentral Lobule L	-1	-35	60	2.917	
Cerebelum 8 R	27	-70	-51	4.281	2236
Cerebelum 8 R	29	-59	-51	3.477	
Parietal Sup R	29	-65	56	4.242	15386
Angular R	25	-61	47	4.230	
Parietal Sup R	31	-70	56	4.220	
Parietal Sup R	22	-65	58	4.137	
Frontal Inf Oper L	-38	8	27	3.903	2651
Frontal Inf Tri L	-40	19	29	3.558	
Precentral L	-36	-4	58	3.891	2491
Precentral L	-40	-2	51	3.794	
Frontal Sup L	-21	-2	47	3.546	
Frontal Mid L	-27	2	51	2.937	
Parietal Sup L	-18	-63	53	3.886	15056
Parietal Sup L	-34	-65	53	3.784	
Parietal Sup L	-27	-68	47	3.765	
Parietal Inf L	-34	-54	51	3.672	
Postcentral L	-21	-48	56	3.869	181
unspecified in AAL	-23	39	-27	3.793	255
unspecified in AAL	-23	-30	9	3.714	1405
unspecified in AAL	-32	-30	7	3.456	
Thalamus L	-10	-13	9	3.452	
Cerebelum 8 L	-29	-70	-51	3.690	1213
Frontal Sup L	-12	11	47	3.672	351
Supp Motor Area L	-1	11	56	3.620	702
unspecified in AAL	-16	-39	65	3.592	276
Calcarine L	-3	-72	7	3.580	819

Frontal Mid R	38	-2	58	3.497	202
Frontal Inf Oper R	42	13	25	3.477	873
ParaHippocampal L	-18	4	-25	3.475	543
Fusiform R	33	-4	-34	3.466	1288
Fusiform R	40	-10	-31	3.364	
Temporal Inf R	62	-39	-23	3.464	212
Supp Motor Area R	14	0	62	3.419	149
Vermis 3	5	-43	-18	3.365	447
unspecified in AAL	3	2	-14	3.326	319
unspecified in AAL	-1	-2	-7	2.862	
Hippocampus L	-16	-6	-14	3.276	468
unspecified in AAL	25	-15	36	3.229	106
Occipital Sup L	-10	-83	47	3.223	170
unspecified in AAL	5	-28	-3	3.069	149
Cerebelum 10 L	-23	-41	-42	3.050	266
Precuneus L	-12	-52	71	2.994	149
Amygdala R	20	0	-12	2.992	181
Calcarine R	20	-52	3	2.990	202
unspecified in AAL	-29	-28	-47	2.875	74
unspecified in AAL	-49	-26	65	2.854	53
Thalamus L	-7	-19	18	2.831	63

Supplementary Table 12 | Recall-related face representation strength. Mixed linear model regression results fit to the probability-scaled likelihood of face-processing during deconvolved single-trial recall epochs during the feedback-based association learning task.

Model:	MixedLM	Dependent variable	Face Probability (Recall)			
Number of observations:	3183	Method:	REML			
Number of groups:	28	Scale:	0.0480			
Minimal group size:	101	Log-Likelihood:	248.0065			
Maximal group size:	120	Converged:	Yes			
Mean group size:	113.7					
	**Coefficient**	**Standard error**	***Z***	***p***	**[0.025**	**0.975]**
**Intercept**	0.664	0.030	22.235	<0.001	0.606	0.723
**Encoding demand**	0.032	0.005	6.894	<0.001	0.023	0.041
**Subsequent recall success**	0.011	0.006	1.922	0.055	-0.000	0.022
**Group variable**	0.024	0.031	-	-	-	-

Supplementary Table 13 | Encoding-related face representation strength. Mixed linear model regression results fit to the probability-scaled likelihood of face-processing during deconvolved single-trial encoding epochs.

Model:	MixedLM	Dependent variable	Face Probability (Encoding)
Number of observations:	3183	Method:	REML
Number of groups:	28	Scale:	0.0654
Minimal group size:	101	Log-Likelihood:	-235.6007
Maximal group size:	120	Converged:	Yes
Mean group size:	113.7		

	**Coefficient**	**Standard error**	***z***	***p***	**[0.025**	**0.975]**
**Intercept**	0.435	0.026	16.423	<0.001	0.383	0.487
**Encoding demand**	0.039	0.005	7.176	<0.001	0.028	0.049
**Subsequent recall success**	0.015	0.007	2.254	0.024	0.002	0.028
**Group variable**	0.019	0.021	-	-	-	-

Supplementary Table 14 | Inter-trial-interval-related face representation strength. Mixed linear model regression results fit to the probability-scaled likelihood of face-processing during deconvolved single-trial inter-trial-interval epochs during the presentation of the fixation cross in the feedback-based association learning task.

Model:	MixedLM	Dependent variable	Face Probability (ITI)			
Number of observations:	3183	Method:	REML			
Number of groups:	28	Scale:	0.0382			
Minimal group size:	101	Log-Likelihood:	606.6500			
Maximal group size:	120	Converged:	Yes			
Mean group size:	113.7					
	**Coefficient**	**Standard error**	***z***	***p***	**[0.025**	**0.975]**
**Intercept**	0.244	0.031	7.752	<0.001	0.182	0.305
**Encoding demand**	0.006	0.004	1.571	0.116	-0.002	0.015
**Subsequent recall success**	-0.002	0.005	-0.343	0.732	-0.012	0.008
**Group variable**	0.027	0.038	-	-	-	-

Supplementary Table 15 | Recall-related face representation strength. Mixed linear model regression results fit to the probability-scaled likelihood of face-processing during deconvolved single-trial recall epochs during the feedback-based association learning task. The analysis is restricted to ErrorError and ErrorCorrect trials.

Model:	MixedLM	Dependent variable	Face Probability (Recall)
Number of observations:	1656	Method:	REML
Number of groups:	28	Scale:	0.0448
Minimal group size:	35	Log-Likelihood:	166.1355
Maximal group size:	102	Converged:	Yes
Mean group size:	59.1		

	**Coefficient**	**Standard error**	***Z***	***p***	**[0.025**	**0.975]**
**Intercept**	0.696	0.032	22.019	<0.001	0.634	0.758
**Subsequent recall success**	0.011	0.006	1.919	0.055	-0.000	0.022
**Group variable**	0.027	0.036	-	-	-	-

Supplementary Table 16 | Encoding-related face representation strength. Mixed linear model regression results fit to the probability-scaled likelihood of face-processing during deconvolved single-trial encoding epochs. The analysis is restricted to ErrorError and ErrorCorrect trials.

Model:	MixedLM	Dependent variable		Face Probability (Encoding)		
Number of observations:	1656	Method:		REML		
Number of groups:	28	Scale:		0.0663		
Minimal group size:	35	Log-Likelihood:		-148.2441		
Maximal group size:	102	Converged:		Yes		
Mean group size:	59.1					
	**Coefficient**	**Standard error**	***z***	***p***	**[0.025**	**0.975]**
**Intercept**	0.475	0.027	17.712	<0.001	0.422	0.527
**Subsequent recall success**	0.015	0.007	2.149	0.032	0.032	0.001
**Group variable**	0.019	0.021	-	-	-	-

Supplementary Table 17 | Inter-trial-interval-related face representation strength. Mixed linear model regression results fit to the probability-scaled likelihood of face-processing during deconvolved single-trial Inter-Trial-Interval epochs during the presentation of the fixation cross in the feedback-based association learning task. The analysis is restricted to ErrorError and ErrorCorrect trials.

Model:	MixedLM	Dependent variable	Face Probability (ITI)
Number of observations:	1656	Method:	REML
Number of groups:	28	Scale:	0.0390
Minimal group size:	35	Log-Likelihood:	278.2743
Maximal group size:	102	Converged:	Yes
Mean group size:	59.1		

	**Coefficient**	**Standard error**	***z***	***p***	**[0.025**	**0.975]**
**Intercept**	0.250	0.032	7.909	<0.001	0.188	0.312
**Subsequent recall success**	-0.001	0.005	-0.279	0.780	-0.012	0.009
**Group variable**	0.027	0.039	-	-	-	-

Supplementary Methods

MRI data preprocessing

Results included in this manuscript come from preprocessing performed using fMRIPrep 23.2.2 (Esteban et al. (2019); Esteban et al. (2018); RRID:SCR_016216), which is based on Nipype 1.8.6 (K. Gorgolewski et al. (2011); K. J. Gorgolewski et al. (2018); RRID:SCR_002502).

Preprocessing of B0 inhomogeneity mappings

A total of 1 fieldmaps were found available within the input BIDS structure for this particular subject. A B0 nonuniformity map (or fieldmap) was estimated from the phase-drift map(s) measure with two consecutive GRE (gradient-recalled echo) acquisitions. The corresponding phase-map(s) were phase-unwrapped with prelude (FSL None).

Anatomical data preprocessing

A total of 1 T1-weighted (T1w) images were found within the input BIDS dataset. The T1w image was corrected for intensity non-uniformity (INU) with N4BiasFieldCorrection (Tustison et al. 2010), distributed with ANTs 2.5.0 (Avants et al. 2008, RRID:SCR_004757), and used as T1w-reference throughout the workflow. The T1w-reference was then skull-stripped with a Nipype implementation of the antsBrainExtraction.sh workflow (from ANTs), using OASIS30ANTs as target template. Brain tissue segmentation of cerebrospinal fluid (CSF), white-matter (WM) and gray-matter (GM) was performed on the brain-extracted T1w using fast (FSL (version unknown), RRID:SCR_002823, Zhang, Brady, and Smith 2001). Volume-based spatial normalization to one standard space (MNI152NLin2009cAsym) was performed through nonlinear registration with antsRegistration (ANTs 2.5.0), using brain-extracted versions of both T1w reference and the T1w template. The following

template was were selected for spatial normalization and accessed with TemplateFlow (23.1.0, Ciric et al. 2022): ICBM 152 Nonlinear Asymmetrical template version 2009c [Fonov et al. (2009), RRID:SCR_008796; TemplateFlow ID: MNI152NLin2009cAsym].

Functional data preprocessing

For each of the 3 BOLD runs found per subject (across all tasks and sessions), the following preprocessing was performed. First, a reference volume was generated, using a custom methodology of fMRIPrep, for use in head motion correction. Head-motion parameters with respect to the BOLD reference (transformation matrices, and six corresponding rotation and translation parameters) are estimated before any spatiotemporal filtering using mcflirt (FSL , Jenkinson et al. 2002). The estimated fieldmap was then aligned with rigid-registration to the target EPI (echo-planar imaging) reference run. The field coefficients were mapped on to the reference EPI using the transform. The BOLD reference was then co-registered to the T1w reference using mri_coreg (FreeSurfer) followed by flirt (FSL , Jenkinson and Smith 2001) with the boundary-based registration (Greve and Fischl 2009) cost-function. Co-registration was configured with six degrees of freedom. Several confounding time-series were calculated based on the preprocessed BOLD: framewise displacement (FD), DVARS and three region-wise global signals. FD was computed using two formulations following Power (absolute sum of relative motions, Power et al. (2014)) and Jenkinson (relative root mean square displacement between affines, Jenkinson et al. (2002)). FD and DVARS are calculated for each functional run, both using their implementations in Nipype (following the definitions by Power et al. 2014). The three global signals are extracted within the CSF, the WM, and the whole-brain masks. Additionally, a set of physiological regressors were extracted to allow for component-based noise correction (CompCor, Behzadi et al. 2007). Principal components are

estimated after high-pass filtering the preprocessed BOLD time-series (using a discrete cosine filter with 128s cut-off) for the two CompCor variants: temporal (tCompCor) and anatomical (aCompCor). tCompCor components are then calculated from the top 2% variable voxels within the brain mask. For aCompCor, three probabilistic masks (CSF, WM and combined CSF+WM) are generated in anatomical space. The implementation differs from that of Behzadi et al. in that instead of eroding the masks by 2 pixels on BOLD space, a mask of pixels that likely contain a volume fraction of GM is subtracted from the aCompCor masks. This mask is obtained by thresholding the corresponding partial volume map at 0.05, and it ensures components are not extracted from voxels containing a minimal fraction of GM. Finally, these masks are resampled into BOLD space and binarized by thresholding at 0.99 (as in the original implementation). Components are also calculated separately within the WM and CSF masks. For each CompCor decomposition, the k components with the largest singular values are retained, such that the retained components' time series are sufficient to explain 50 percent of variance across the nuisance mask (CSF, WM, combined, or temporal). The remaining components are dropped from consideration. The head-motion estimates calculated in the correction step were also placed within the corresponding confounds file. The confound time series derived from head motion estimates and global signals were expanded with the inclusion of temporal derivatives and quadratic terms for each (Satterthwaite et al. 2013). Frames that exceeded a threshold of 0.5 mm FD or 1.5 standardized DVARS were annotated as motion outliers. Additional nuisance timeseries are calculated by means of principal components analysis of the signal found within a thin band (crown) of voxels around the edge of the brain, as proposed by (Patriat, Reynolds, and Birn 2017). All resamplings can be performed with a single interpolation step by composing all the

pertinent transformations (i.e. head-motion transform matrices, susceptibility distortion correction when available, and co-registrations to anatomical and output spaces). Gridded (volumetric) resamplings were performed using nitransforms, configured with cubic B-spline interpolation.

Many internal operations of fMRIPrep use Nilearn 0.10.2 (Abraham et al. 2014, RRID:SCR_001362), mostly within the functional processing workflow. For more details of the pipeline, see the section corresponding to workflows in fMRIPrep's documentation.

Copyright Waiver

The above boilerplate text was automatically generated by fMRIPrep with the express intention that users should copy and paste this text into their manuscripts unchanged. It is released under the CC0 license.

Supplementary References

Abraham, Alexandre, Fabian Pedregosa, Michael Eickenberg, Philippe Gervais, Andreas Mueller, Jean Kossaifi, Alexandre Gramfort, Bertrand Thirion, and Gael Varoquaux. 2014. "Machine Learning for Neuroimaging with Scikit-Learn." *Frontiers in Neuroinformatics 8*. https://doi.org/10.3389/fninf.2014.00014.

Avants, B. B., C. L. Epstein, M. Grossman, and J. C. Gee. 2008. "Symmetric Diffeomorphic Image Registration with Cross-Correlation: Evaluating Automated Labeling of Elderly and Neurodegenerative Brain." *Medical Image Analysis 12* (1): 26–41. https://doi.org/10.1016/j.media.2007.06.004.

Behzadi, Yashar, Khaled Restom, Joy Liau, and Thomas T. Liu. 2007. "A Component Based Noise Correction Method (CompCor) for BOLD and Perfusion Based fMRI." *NeuroImage 37* (1): 90–101. https://doi.org/10.1016/j.neuroimage.2007.04.042.

Ciric, R., William H. Thompson, R. Lorenz, M. Goncalves, E. MacNicol, C. J. Markiewicz, Y. O. Halchenko, et al. 2022. “TemplateFlow: FAIR-Sharing of Multi-Scale, Multi-Species Brain Models.” *Nature Methods 19*: 1568–71. https://doi.org/10.1038/s41592-022-01681-2.

Esteban, Oscar, Ross Blair, Christopher J. Markiewicz, Shoshana L. Berleant, Craig Moodie, Feilong Ma, Ayse Ilkay Isik, et al. 2018. “fMRIPrep 23.2.2.” *Software*. https://doi.org/10.5281/zenodo.852659.

Esteban, Oscar, Christopher Markiewicz, Ross W Blair, Craig Moodie, Ayse Ilkay Isik, Asier Erramuzpe Aliaga, James Kent, et al. 2019. “fMRIPrep: A Robust Preprocessing Pipeline for Functional MRI.” *Nature Methods 16*: 111–16. https://doi.org/10.1038/s41592-018-0235-4.

Fonov, VS, AC Evans, RC McKinstry, CR Almli, and DL Collins. 2009. “Unbiased Nonlinear Average Age-Appropriate Brain Templates from Birth to Adulthood.” *NeuroImage 47*, Supplement 1: S102. https://doi.org/10.1016/S1053-8119(09)70884-5.

Gorgolewski, K., C. D. Burns, C. Madison, D. Clark, Y. O. Halchenko, M. L. Waskom, and S. Ghosh. 2011. “Nipype: A Flexible, Lightweight and Extensible Neuroimaging Data Processing Framework in Python.” *Frontiers in Neuroinformatics 5*: 13. https://doi.org/10.3389/fninf.2011.00013.

Gorgolewski, Krzysztof J., Oscar Esteban, Christopher J. Markiewicz, Erik Ziegler, David Gage Ellis, Michael Philipp Notter, Dorota Jarecka, et al. 2018. “Nipype.” *Software*. https://doi.org/10.5281/zenodo.596855.

Greve, Douglas N, and Bruce Fischl. 2009. “Accurate and Robust Brain Image Alignment Using Boundary-Based Registration.” *NeuroImage 48* (1): 63–72. https://doi.org/10.1016/j.neuroimage.2009.06.060.

Jenkinson, Mark, Peter Bannister, Michael Brady, and Stephen Smith. 2002. “Improved Optimization for the Robust and Accurate Linear Registration and Motion Correction of Brain Images.” *NeuroImage 17* (2): 825–41. https://doi.org/10.1006/nimg.2002.1132.

Jenkinson, Mark, and Stephen Smith. 2001. “A Global Optimisation Method for Robust Affine Registration of Brain Images.” Medical Image Analysis 5 (2): 143–56. https://doi.org/10.1016/S1361-8415(01)00036-6.

Patriat, Rémi, Richard C. Reynolds, and Rasmus M. Birn. 2017. “An Improved Model of Motion-Related Signal Changes in fMRI.” *NeuroImage 144*, Part A (January): 74–82. https://doi.org/10.1016/j.neuroimage.2016.08.051.

Power, Jonathan D., Anish Mitra, Timothy O. Laumann, Abraham Z. Snyder, Bradley L. Schlaggar, and Steven E. Petersen. 2014. “Methods to Detect, Characterize, and Remove Motion Artifact in Resting State fMRI.” *NeuroImage 84* (Supplement C): 320–41. https://doi.org/10.1016/j.neuroimage.2013.08.048.

Satterthwaite, Theodore D., Mark A. Elliott, Raphael T. Gerraty, Kosha Ruparel, James Loughead, Monica E. Calkins, Simon B. Eickhoff, et al. 2013. “An improved framework for confound regression and filtering for control of motion artifact in the preprocessing of resting-state functional connectivity data.” *NeuroImage 64* (1): 240–56. https://doi.org/10.1016/j.neuroimage.2012.08.052.

Tustison, N. J., B. B. Avants, P. A. Cook, Y. Zheng, A. Egan, P. A. Yushkevich, and J. C. Gee. 2010. “N4ITK: Improved N3 Bias Correction.” *IEEE Transactions on Medical Imaging 29* (6): 1310–20. https://doi.org/10.1109/TMI.2010.2046908.

Zhang, Y., M. Brady, and S. Smith. 2001. “Segmentation of Brain MR Images Through a Hidden Markov Random Field Model and the Expectation-Maximization

Algorithm." *IEEE Transactions on Medical Imaging 20* (1): 45–57. https://doi.org/10.1109/42.906424.
