# Supplementary material for: Error-driven upregulation of memory representations
Source: Commun Psychol. 2025 Jan 30;3:17. doi: 10.1038/s44271-025-00199-5 (PMC11782628; doi:10.1038/s44271-025-00199-5)
Supplement: Supplementary file 3 — Reporting Summary [file 44271_2025_199_MOESM3_ESM.pdf]

nature portfolio

Corresponding author(s): Alexander Weuthen

Last updated by author(s): Dec 17, 2024

Reporting Summary

Nature Portfolio wishes to improve the reproducibility of the work that we publish. This form provides structure for consistency and transparency in reporting. For further information on Nature Portfolio policies, see our Editorial Policies and the Editorial Policy Checklist.

Statistics

For all statistical analyses, confirm that the following items are present in the figure legend, table legend, main text, or Methods section.

n/a	Confirmed	
☐	☒	The exact sample size (n) for each experimental group/condition, given as a discrete number and unit of measurement
☐	☒	A statement on whether measurements were taken from distinct samples or whether the same sample was measured repeatedly
☐	☒	The statistical test(s) used AND whether they are one- or two-sided<br>*Only common tests should be described solely by name; describe more complex techniques in the Methods section.*
☐	☒	A description of all covariates tested
☐	☒	A description of any assumptions or corrections, such as tests of normality and adjustment for multiple comparisons
☐	☒	A full description of the statistical parameters including central tendency (e.g. means) or other basic estimates (e.g. regression coefficient) AND variation (e.g. standard deviation) or associated estimates of uncertainty (e.g. confidence intervals)
☐	☒	For null hypothesis testing, the test statistic (e.g. F, t, r) with confidence intervals, effect sizes, degrees of freedom and P value noted<br>*Give P values as exact values whenever suitable.*
☒	☐	For Bayesian analysis, information on the choice of priors and Markov chain Monte Carlo settings
☒	☐	For hierarchical and complex designs, identification of the appropriate level for tests and full reporting of outcomes
☐	☒	Estimates of effect sizes (e.g. Cohen's d, Pearson's r), indicating how they were calculated

Our web collection on statistics for biologists contains articles on many of the points above.

Software and code

Policy information about availability of computer code

Data collection	Data were collected using Matlab and experimental paradigms were presented using Psychophysics Toolbox 3. Magnetic resonance imaging data were obtained by a 3 Tesla Siemen Prisma scanner.
Data analysis	MRI data were converted using dcm2niix (version v1.0.20190902). For preprocessing, fMRIPrep (version 23.2.2) was run with a singularity image (version 2.6.1-dist) wrapped around a docker container. Behavioral and fMRI analyses were based on custom Python code within Jupyter Lab, using plotting functions from Matplotlib and Seaborn, numerical processing and statistical testing with Numpy, Scipy, Pandas and Statsmodels, and decoding tools from Scikitlearn and Nilearn (version 0.10.0). Visualization of fMRI results was based on MRIcroGL (version 1.2.20220720b).

For manuscripts utilizing custom algorithms or software that are central to the research but not yet described in published literature, software must be made available to editors and reviewers. We strongly encourage code deposition in a community repository (e.g. GitHub). See the Nature Portfolio guidelines for submitting code & software for further information.

Data

Policy information about availability of data

All manuscripts must include a data availability statement. This statement should provide the following information, where applicable:

- Accession codes, unique identifiers, or web links for publicly available datasets
- A description of any restrictions on data availability
- For clinical datasets or third party data, please ensure that the statement adheres to our policy

Publicly available images of emotionally neutral faces from the Picture Database of Morphed Faces (Jäger et al., 2005) and house images from the DalHouses sample (Filliter et al., 2016) were used. Unthresholded statistical maps will be uploaded on public repositories.

Human research participants

Policy information about studies involving human research participants and Sex and Gender in Research.

Reporting on sex and gender	15 male and 15 female participants according to self-reported sex
Population characteristics	Age between 18 and 35 (mean = 24.73, standard deviation = 4.53). No data on race/ethnicity collected. No history of psychiatric or neurological disorders.
Recruitment	Participants were recruited trough an existing local database. Inclusion criteria were checked via phone interview.
Ethics oversight	The study was approved by the ethics committee of the medical faculty at Otto-von-Guericke University Magdeburg, Germany.

Note that full information on the approval of the study protocol must also be provided in the manuscript.

Field-specific reporting

Please select the one below that is the best fit for your research. If you are not sure, read the appropriate sections before making your selection.

☐ Life sciences ☒ Behavioural & social sciences ☐ Ecological, evolutionary & environmental sciences

For a reference copy of the document with all sections, see nature.com/documents/nr-reporting-summary-flat.pdf

Behavioural & social sciences study design

All studies must disclose on these points even when the disclosure is negative.

Study description	In this study, we recorded quantitative experimental fMRI and behavioral data of 30 participants while they performed a novel feedback-based association learning task and 1-back localizer task.
Research sample	Young adults (15 male,15 female) participated in the study. They were compensated with money (10 EUR per hour) or with study credits for their time.
Sampling strategy	Participants were selected according to the following requirements: age between 18 and 35, body mass index between 20 and 30 kg/m2, non-smokers, no history of psychiatric or neurological disorders, no metal implants.
Data collection	Data were recorded from a 3T Siemens fMRI scanner and via Matlab/Psychtoolbox.
Timing	Data were recorded between Dec 2019 and Dec 2020.
Data exclusions	Two participants were excluded from fMRI analyes in the 1-back localizer task and follow-up analyses, because an adequate understanding of and sufficient attention to the 1-back task could not be ensured due to the poor performance.
Non-participation	No participants declined participation or dropped out.
Randomization	Participants were presented with the stimuli in a pseudo-randomized order.

Reporting for specific materials, systems and methods

We require information from authors about some types of materials, experimental systems and methods used in many studies. Here, indicate whether each material, system or method listed is relevant to your study. If you are not sure if a list item applies to your research, read the appropriate section before selecting a response.

Materials & experimental systems

n/a	Involved in the study
☒	☐ Antibodies
☒	☐ Eukaryotic cell lines
☒	☐ Palaeontology and archaeology
☒	☐ Animals and other organisms
☒	☐ Clinical data
☒	☐ Dual use research of concern

Methods

n/a	Involved in the study
☒	☐ ChIP-seq
☒	☐ Flow cytometry
☐	☒ MRI-based neuroimaging

Magnetic resonance imaging

Experimental design

Design type

Event-related fMRI design.

Design specifications

Each participant performed four blocks and five run of the two tasks (FALT, Localizer). In FALT, there were 8 trials per block, resulting in 160 trials in total. In the Localizer, there were 4 stimuli per block, resulting in 80 trials in total.

Behavioral performance measures

In FALT, trial types were differentiated depending on memory recall success in the current and subsequent trials, and based on the selected confidence level. In the Localizer, trial types were differentiated based on signal detection theory (hit, miss, correct rejection, false alarm).

Acquisition

Imaging type(s)

functional and structural MRI

Field strength

3 Tesla

Sequence & imaging parameters

Structural MRI data were assessed using a magnetization prepared rapid gradient echo sequence in sagittal slices (voxel size = 1 x 1 x 1 mm, matrix size = 192 x 256 x 256, repetition time = 2.5 s, echo time = 0.00282 s, flip angle = 7°, multi band factor = 2).
Functional MRI scans were recorded with a field of view aligned to anterior and posterior commissures (voxel size = 2.2 x 2.2 x 2.2 mm, matrix size = 100 x 100 x 66, repetition time = 2.0 s, echo time = 0.03 s, flip angle = 80°, multi band factor = 2, interleaved order, no interslice gap).

Area of acquisition

Whole brain scans.

Diffusion MRI ☐ Used ☒ Not used

Preprocessing

Preprocessing software

Preprocessing with fMRIPrep (version 23.2.2) encompassed slice time correction, susceptibility distortion correction, boundary-based registration and spatial normalization to obtain images in MNI152NLin2009cAsym output space, keeping the size of 2.2 mm3 voxels.

Normalization

Data were normalized within the fMRIPrep workflow. For the structural images, volume-based spatial normalization to standard space (MNI152NLin2009cAsym) was performed through nonlinear registration with antsRegistration. a reference volume was generated, using a custom methodology of fMRIPrep, for use in head motion correction. Head-motion-parameters with respect to the BOLD reference (transformation matrices, and six corresponding rotation and translation are estimated before any spatiotemporal filtering using mcflirt. The estimated fieldmap was then aligned with rigid-registration to the target echo-planar imaging reference run. The field coefficients were mapped on to the reference EPI using the transform. The BOLD reference was then co-registered to the T1w reference using mri_coreg ollowed by flirt with the boundary-based registration cost-function. Co-registration was configured with six degrees of freedom.

Normalization template

MNI152NLin2009cAsym, 2.2mm3 resolution

Noise and artifact removal

For simultaneous denoising and fitting of event-related hemodynamic response functions, GLMs on the preprocessed images contained following confound regressors:24 motion parameters (six rigid body motion parameters, six derivatives, and respective twelve squared motion parameters), 18 physiological regressors (six cardiac, eight respiration, four combined cardiac and respiration), ten anatomical component correction regressors (five white matter, five cerebrospinal fluid), the global signal, a cosine drift model and a constant intercept.

Volume censoring

Frames that exceeded a threshold of 0.5 mm FD or 1.5 standardized DVARS were annotated as motion outliers and used for volume censoring.

Statistical modeling & inference

Model type and settings

Mass-univariate GLM fMRI analyses were conducted by simultaneously fitting a hemodynamic response function using the Glover model convolved with respective event regressors in both tasks. Upon statistical testing of the group results in a second level GLM, contrasts maps were smoothed with an 8 mm kernel and a voxel-wise false-discovery rate threshold was applied, removing clusters with an extent of less than five continuous voxels (equivalent to clusters of at least 53.24 mm3). Multivariate cross-classification was trained on the 1-back task and applied to FALT. In a third mass-univariate GLM, neurophysiological correlates of the predicted class-probability were assessed and contrast maps were calculated according to the second level analyses described above.

Effect(s) tested

GLM 1: FALT error monitoring (I: failed low confident recall > successful high confident recall, II: low > high confidence, III: negative > positive feedback) and FALT error-subsequent memory effect (lErrorLowConfident_ErrorLowConfident)
GLM 2: Face-processing (FaceCorrectRejection > HouseCorrectRejection) and house processing HouseCorrectRejection) > (FaceCorrectRejection)
GLM 3: Multivariate class probability (parametric + constant)

Specify type of analysis: ☐ Whole brain ☐ ROI-based ☒ Both

Anatomical location(s): Cytoarchitectonic probability masks for the fusiform gyrus derived from the Julich Brain Atlas (Eickhoff et al., 2005).

Statistic type for inference (See Eklund et al. 2016): voxel-wise

Correction: FDR

Models & analysis

n/a	Involved in the study
☒	☐ Functional and/or effective connectivity
☒	☐ Graph analysis
☐	☒ Multivariate modeling or predictive analysis

Multivariate modeling and predictive analysis

The GLMs were adapted for single-trial deconvolution according to the least-squares separate approach. The 1-back localizer task was used to train a model on predicting face-processing using a balanced probability-scaled support vector machine (C=1) with a squared penalty function. Within the five-fold leave-one-run-out cross-validation, a standard scaler (M = 0, SD = 1) was fit to the four training runs and applied to the left-out run. Univariate feature selection was applied by maintaining only the beta-weights of the 14 voxels with the strongest positive ANOVA effects, to obtain results for participant-specific FFA voxels and to reach a feature-to-sample ratio of approximately 1:5 before fitting the support vector machine. Decoding accuracies were evaluated by testing whether the average accuracies of the five runs per participant exceeded a chance level of 50 % with a one-sample t-test. Face and house trials were tested for equal decoding accuracies with a t-test for dependent samples to ensure that the FFA-based face-processing model was balanced and did not prefer either of the two categories. After leave-one-run-out cross-validated model evaluation, trials from all five folds were included in model training. A full model was fit on correct rejection trials of all localizer task runs of a participant with the same scaling procedure and feature selection as during cross-validation. The model then predicted the probability for face-processing during memory-relevant epochs in FALT, which was used for GLM3 and follow-up behavioral analyses.
